# The use of extracorporeal shock wave therapy for the treatment of bone marrow oedema — a systematic review and meta-analysis

**DOI:** 10.1186/s13018-021-02484-5

**Published:** 2021-06-09

**Authors:** Jonathan Häußer, Juliane Wieber, Philip Catalá-Lehnen

**Affiliations:** 1 LANS Medicum Hamburg — Center for Sports and Regenerative Medicine, Stephansplatz 5, 20354 Hamburg, Germany; 2 LANS Medicum Hamburg — Center for Sports and Regenerative Medicine, Hohe Bleichen 24/26, 20354 Hamburg, Germany

**Keywords:** Bone marrow oedema, Shock wave therapy, Conservative therapy, Non-invasive treatment, Total hip arthroplasty, Non-traumatic osteonecrosis

## Abstract

**Background:**

Extracorporeal shock wave therapy (ESWT) has been used for various pathologies associated with bone marrow oedema (BME). However, it is still not clear whether ESWT may be favourable in the treatment of BME. Therefore, the aim of this systematic review was to assess the efficacy of ESWT for the treatment of BME.

**Methods:**

MEDLINE was searched for relevant literature with no time constraints. Both randomized and non-randomized trials were included. Case reports and conference abstracts were excluded. Titles and abstracts were screened and full-text articles of included studies were retrieved. Data on the effect of ESWT on pain, function, and the BME area on magnet resonance imaging were extracted.

**Results:**

Pain, function, and magnet resonance imaging results all improved across the studies — regardless of whether it was a randomized or non-randomized study. This effect was consistent across multiple pathologies such as osteonecrosis of the femoral head, BME associated with knee osteoarthritis, Kienböck’s disease, and osteitis pubis. The meta-analysis showed that pain (after 1 month: weighted mean difference (WMD) = − 2.23, 95% CI − 2.58 to − 1.88, *P* < 0.0001; after 3–6 month: WMD = − 1.72, 95% CI − 2.52 to − 0.92, *P* < 0.00001) and function (after 1 month: WMD = − 1.59, 95% CI − 2.04 to − 1.14, *P* < 0.0001; after 3–6 month: WMD = − 2.06, 95% CI − 3.16 to − 0.96, *P* = 0.0002; after ≥ 12 month: WMD = − 1.20, 95% CI − 1.83 to − 0.56, *P* = 0.0002) was reduced in terms of ESWT treatment compared to a control group.

**Conclusions:**

Based on the available evidence, ESWT may be an adequate option for conservative therapy in pathologies involving BME.

**Trial registration:**

PROSPERO, CRD42021201719. Registered 23 December 2020

**Supplementary Information:**

The online version contains supplementary material available at 10.1186/s13018-021-02484-5.

## Introduction

Bone marrow oedema (BME) may occur in many different locations. While the exact pathogenesis is still being debated, BME presents as a painful increase in interstitial fluid [1]. It is most likely a vascular reaction to external or internal disorders [2].

BME can be categorized as primary or secondary. While secondary BME is due to another underlying diagnosis, the cause of primary BME is not clear [1]. Primary BME usually affects the hip, knee, ankle, or foot. Secondary BME is most often due to trauma [3] but imaging findings in osteoarthritis of the knee and avascular osteonecrosis of the femoral head include BME as well. In knee osteoarthritis, BME is usually painful. Subchondral oedema may even indicate a quick structural progression. The oedema may be caused by increased mechanical load or altered weight bearing [4]. Osteonecrosis of the femoral head affects about 20,000 patients in the USA each year [5]. About half of them progress to collapse if left untreated and need total hip arthroplasty [5]. BME most commonly occurs in young women and middle-aged men with men being affected three times as often [3].

It is widespread that symptoms and imaging findings may take 3–18 months to resolve [1, 6]. Initial treatment is usually symptomatic including limited weight bearing, analgesics, and physical therapy [1, 6]. Additionally, corticosteroids, bisphosphonates, and prostaglandin inhibitors such as iloprost have been used. Surgery is the last resort and the most common surgical technique is core decompression. This usually leads to significant improvements after 4 weeks [7]. However, surgery is prone to complications such as wound infections, fracture, and haematoma [1, 6]. With BME being a self-limiting condition, some consider surgery too invasive.

Extracorporeal shock wave therapy (ESWT) is another option for conservative treatment. A growing body of evidence has shown its effectiveness in multiple orthopaedic conditions. Among others, ESWT has been proven to be effective in avascular necrosis of the femoral head. Reductions in the extent of BME have been observed in early stages of the disease after shockwave treatment [6].

The mechanism of action is not entirely clear. Generally speaking, shock wave therapy promotes a tissue’s self-healing capabilities [8]. In bone tissue, this involves stimulation of osteoblasts and periosteal cells, differentiation of stem cells, and increased secretion of nitric oxide synthase and vascular endothelial growth factor thus leading to increased neovascularization [1, 7]. Additionally, the periosteum is stimulated and osteoclasts activity is reduced [8].

Although BME is usually self-limiting, conservative therapy has not been very successful in shortening the usual course of the disease. Surgery on the contrary is very invasive and comes with the risk of several complications [1]. This calls for more successful conservative measures. We therefore reviewed the literature on the effectiveness of ESWT in the treatment of BME. The question of this review was whether shock wave therapy is an effective treatment for improving pain and function in patients displaying BME and whether the effect of shock wave therapy is comparable to other conservative measures and surgery. We report outcomes of pain, function, and MRI in patients with BME after being treated with ESWT.

## Methods

This systematic review and meta-analysis was registered online in PROSPERO (registration number CRD42021201719, see Additional file 3) and was performed following the guidelines of the PRISMA statement. The protocol and the PRISMA checklist were provided as Additional files 1 and 2, respectively. The literature search was performed on MEDLINE on July 6, 2020, using the following search term:

(shock AND wave) AND (bone AND (marrow OR (edema OR oedema)))

**Fig. 1:**
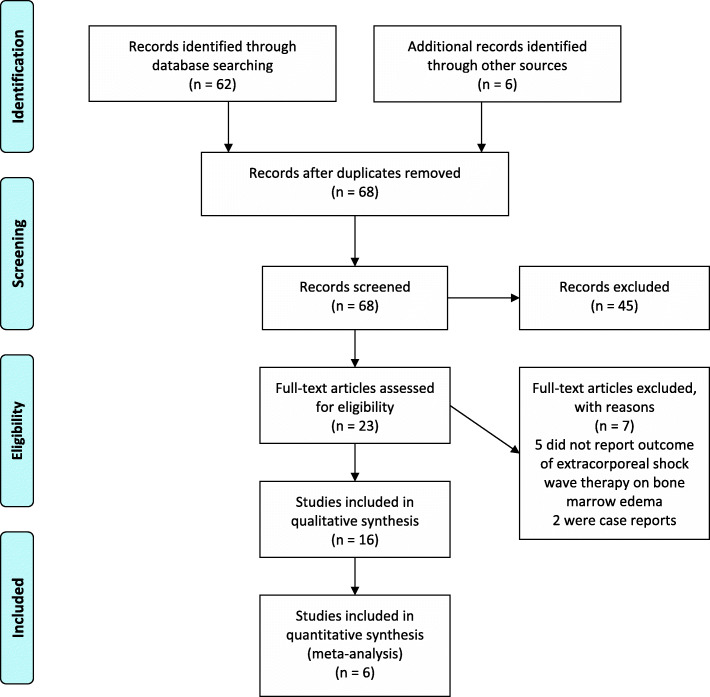
PRISMA flow chart

Titles and abstracts were screened independently by two authors for relevant publications. Articles that reported pain and functional outcomes in the short-, mid-, and long-term as well as changes on MRI were included. Case reports, conference abstracts, and publications not written in English or German were excluded. Of all the remaining publications, full texts were retrieved. If there was disagreement about the inclusion of certain studies, the situation was resolved by consensus. Data on pain, patient reported function, and changes on MRI imaging were extracted independently by the two authors from included papers. The findings are reported according to the PRISMA statement [9]. The flow chart can be found in Fig. 1, for the PRISMA checklist see Additional file 2.

For the assessment of study quality, we used a modified version of the Downs and Black checklist [10] as shown by Korakakis et al. [11]. The modified Downs and Black checklist comprises 27 items with a maximum possible score of 28. The modified version rates item 27 with a maximum score of 1 based on whether power calculation was performed or not. The original version rates item 27 on a scale from 0 to 5 according to a range of study powers [10]. Two independent reviewers assessed methodological quality and disagreements were resolved by a third reviewer.

### Statistical analysis

**Fig. 2:**
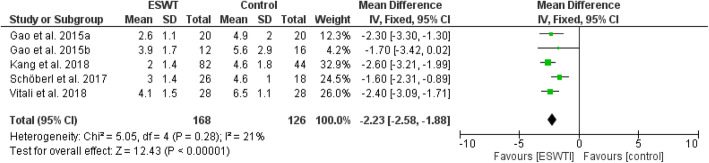
Forest plot comparing the pain outcome after ESWT treatment (short-term; 1 month)

**Fig. 3:**
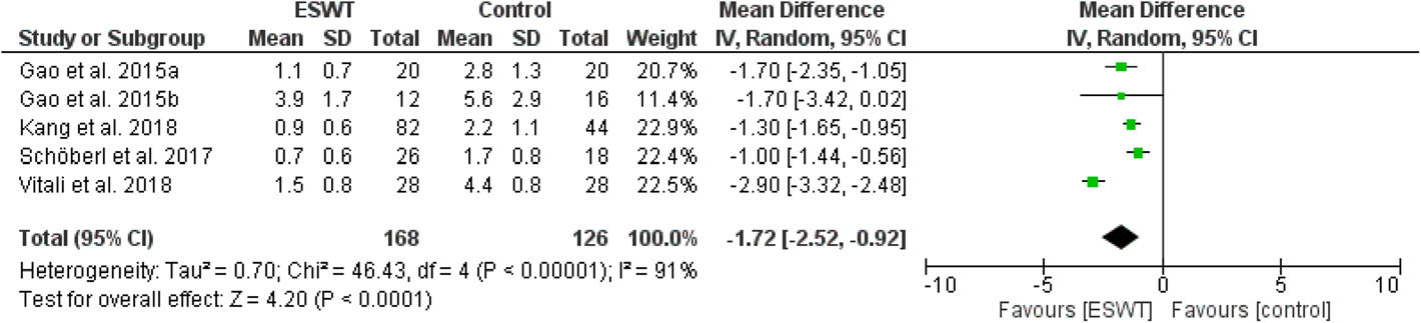
Forest plot comparing the pain outcome after ESWT treatment (mid-term; 3-6 months)

**Fig. 4:**
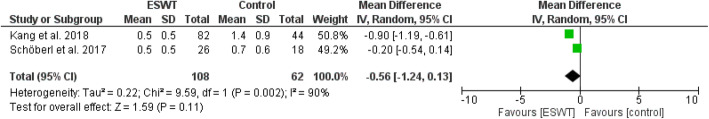
Forest plot comparing the pain outcome after ESWT treatment (long-term; ≥ 12 months)

**Fig. 5:**
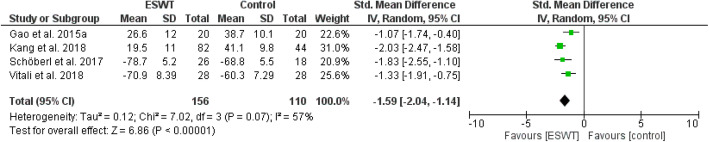
Forest plot comparing the functional outcome after ESWT treatment (short-term; 1 month)

**Fig. 6:**
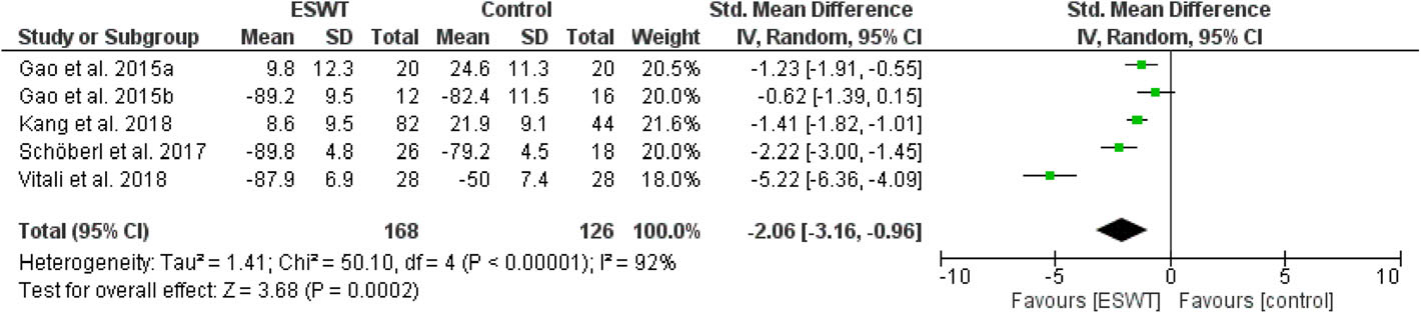
Forest plot comparing the functional outcome after ESWT treatment (mid-term; 3–6 months)

**Fig. 7:**
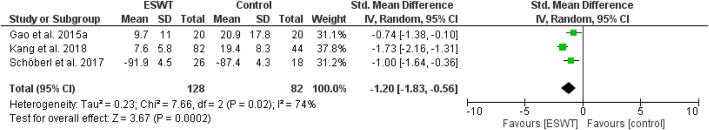
Forest plot comparing the functional outcome after ESWT treatment (long-term; ≥ 12 months)

Review Manager 5.4.1 was adopted for the analysis of included literatures data and a P value of < 0.05 in the data was defined as statistically significant. All data were continuous variables and were applied using weighted mean difference (WMD) and 95% CI. Mean differences were used for outcomes of pain (VAS), and standard mean differences were used for outcomes of function in order to enable comparison across different patient reported outcomes (WOMAC, HOOS, HHS, KSS). Due to methodological heterogenity in the analysed studies, a random effects model was used in most analysis. Data were illustrated by Forest plots (Figs. 2, 3, 4, 5, 6, and 7)

## Results

The search identified 62 publications. After screening, 45 articles were excluded. For all other articles, full texts were retrieved. After reading the full texts, 7 additional articles were excluded due to not reporting outcomes of ESWT or not dealing with BME or being case reports. The remaining 10 documents were included for data synthesis. Additionally, 6 reports were identified by reviewing the references of the included studies (Fig. 1). A summary of the studies which met eligibility criteria is provided as Additional file 1.

### Bone marrow oedema syndrome and osteoarthritis of the knee

There was only one randomized-controlled trial on the treatment of BME of the knee. Gao et al. [1] randomly allocated 40 participants to 2 groups either receiving 2 sessions of shock wave therapy or medical treatment with alprostadil and alendronate. There were greater and more rapid improvements in pain on the VAS and on the Western Ontario and McMaster Universities Osteoarthritis Index (WOMAC) for those treated with shock wave therapy. Also, more pronounced reductions of BME area were found on MRI after 6 months [1].

Additionally, there were three retrospective case series with control groups. Vitali et al. [3] and Sansone et al. [6] reported outcomes of 3 sessions of shock wave treatment in patients with BME of the medial condyle of the knee. While Vitali et al. performed shock wave therapy at weekly intervals, Sansone et al. did so every 3 weeks. Both found greater and more rapid improvements in VAS and Knee Society Score or WOMAC score compared to a control group who received analgesics and partial weight bearing only. Also, the reduction of BME area on MRI was significantly greater after shock wave treatment [3, 6]. A last study included 126 patients with mild to moderate osteoarthritis of the knee having BME. Both groups received alprostadil 10 μg once daily for 2 weeks. One group additionally received two sessions of ESWT, the other group 70 mg oral alendronate once weekly. Pain and WOMAC score improved quicker and to a greater extent in the group receiving ESWT. Improvements in BME on MRI were seen in 90.2% of patients treated with ESWT compared to 61.4% of patients treated with alendronate after 3 months [4].

The general procedure was two to three sessions of ESWT once a week or once every third week with 2000–4000 impulses. They were applied at 2–4 Hz with an energy flux density of 0.22–0.55 mJ/mm^2^.

### Osteonecrosis of the femoral head

There were no randomized-controlled trials of the treatment of osteonecrosis of the femoral head with ESWT. However, two studies were pseudo-randomized, one by medical record number [12] and one by date of treatment [13]. Hsu et al. [12] compared shock wave therapy alone to so called cocktail therapy, which additionally included hyperbaric oxygen therapy and oral alendronate for 12 months. Nevertheless, they found no significant differences in the outcome between the groups [12]. Similarly, the combined treatment with ESWT and alendronate was not more effective than ESWT alone [13].

In a prospective study, patients with avascular necrosis of femoral head ARCO stage I showed rapid improvements in VAS and Harris hip score (HHS); stage II patients also improved. However, this took more time. Finally, outcome in stage III patients was less favourable. Ten out of 15 decided to have total hip arthroplasty (THA) [14].

Chen et al. retrospectively reported a series of 17 patients with bilateral osteonecrosis of the femoral head. On the more severe side, they were treated with THA. The less severe hip was treated with ESWT. The time between treatment of both sides was 17.3 months on average. Patients were generally more satisfied with shock wave treatment. ESWT yielded greater improvement in VAS and HHS and better functional outcome. On the hip treated with THA, 47% suffered from thigh pain and decreased range of motion [15].

Two more retrospective case series reported results after ESWT in non-traumatic osteonecrosis of the femoral head. Gao et al. showed significant improvements of VAS and HHS at 12 months’ follow-up after two sessions of shock wave therapy. There were also significant reductions in BME after treatment [16]. Xie et al. analysed long-term outcomes 10 years after a single shock wave treatment in patients with non-traumatic osteonecrosis of the femoral head. They found significant improvements in Harris Hip score and VAS compared to pre-treatment scores and concluded that shock wave therapy in early stage osteonecrosis of the femoral head is also effective in the longer term [5].

### Bone marrow oedema syndrome of the hip

Two retrospective studies evaluated the outcomes of shock wave treatment in BME syndrome of the hip. In the study by Gao et al. [7], 46 patients received at least 3 months of conservative therapy including limited weight bearing, nonsteroidal anti-inflammatory drugs, alendronate, and alprostadil. Subsequently they either had shock wave therapy or surgical core decompression of the femoral head. All patients recovered within 12 weeks and MRIs after 6 months showed no further pathologic findings. Improvements in VAS were significantly greater for those treated with shock wave therapy and they resumed daily activities sooner. Additionally, full resolution of symptoms occurred significantly earlier after shock wave treatment.

D’Agostino et al. [2] studied 20 subjects with BME syndrome of the hip who received 2 sessions of shock wave treatment. There were large improvements in HHS after 2 months and further significant improvements for every follow-up until 6 months. BME area more than halved after 2 months.

### Osteitis pubis

Schöberl et al. studied athletes with groin pain who displayed BME of the pubic bone also known as osteitis pubis. Forty-four athletes were randomly assigned to be treated with shock wave therapy or to receive a sham treatment. Both groups underwent an intensive rehabilitation programme. VAS and Hip disability and Osteoarthritis Outcome Score showed significantly greater improvements in the shock wave group after 1 and 3 months and this group was able to return to football significantly earlier (73.2 vs. 102.6 days). MRI findings did not differ significantly [17].

### Kienböck’s disease

Kienböck’s disease is another pathology with avascular necrosis of the lunate. As in other entities, a BME usually accompanies complaints in patients. A case series with 22 patients showed that shock wave treatment was effective in improving pain and range of motion after 60 days. BME showed noticeable reduction on MRI post treatment [18].

### Plantar fasciitis

Maki et al. [19] reported a case series of 23 patients with refractory plantar fasciitis after 3 months of conservative therapy. They received 1 or 2 sessions of shock wave therapy and were followed up after 6 months. There were significant improvements in VAS and the Japanese Society for Surgery of the Foot ankle-hindfoot scale. Also a reduction of BME was seen. Before treatment BME of the calcaneus was seen in 11 feet compared to 4 feet after shock wave treatment.

### Adverse events

No major adverse events were reported following ESWT. Only minor side effects like bruising or swelling were observed in the included studies.

### Quantitative synthesis

Figures 2, 3, and 4 show pain outcomes in the analysed studies. We found that pain improved in all analysed study groups receiving shock wave therapy compared to control groups. There was a statistically significant superiority for both short-term, as well as mid-term, results. Only long-term outcome for pain did not reach statistical significance. Similar observations could be made for patient reported function (Figs. 5, 6, and 7). At all follow-up time points, there were larger improvements in patients receiving ESWT. Even though data for WOMAC scores were presented in the study of Sansone et al. [6] this study was excluded from meta-analysis because of contradictory data. While reporting higher WOMAC scores in the ESWT group, they reported a superiority of ESWT. However, higher WOMAC scores generally show worse function [20]. Due to limited data a meta-analysis of radiological outcomes was not possible. Table 1 shows the results of follow-up imaging in study groups receiving ESWT compared to those who did not.

**Table 1:** Changes on MRI after ESWT compared to no ESWT

Study	ESWT	No ESWT	Between group difference
Gao et al. [1]	After 6 months: Reduction in 35% Complete regression in 65% After 12 months: Complete regression in 100%	After 6 months: Reduction in 40% Complete regression in 25% After 12 months: Complete regression in 90%	Significant superiority of ESWT after 6 months (*p* = 0.018)
Schöberl et al. [17]	After 3 months: Grading 1.5	After 3 months: Grading 1.5	n.s.
Kang et al. [4]	After 3 months: Reduction in 90.2% of patients After 6 months: Reduction in 95.1% of patients After 12 months: Reduction in 100% of patients	After 3 months: Reduction in 61.4% of patients After 6 months: Reduction in 79.5% of patients After 12 months: Reduction in 97.7% of patients	After 3 months: Significant superiority of ESWT (*p* < 0.001) After 6 months: Significant superiority of ESWT (*p* = 0.006) After 12 months: n.s.
Sansone et al. [6]	After 6 months: 88% reduction	After 6 months: 41% reduction	Significant superiority of ESWT after 6 months (*p* < 0.001)
Vitali et al. [3]	After 4 months: 77% reduction	After 4 months: 40% reduction	Significant superiority of ESWT after 6 months (*p* < 0.00001)

*ESWT* extracorporeal shock wave therapy, *n.s*. not significant

### Risk of bias

The risk of bias differed widely across the included studies. This was most likely due to the differing designs of the studies since both randomized and non-randomized studies were included. There were only few high-quality studies on this topic and most studies were of retrospective nature. The scores of the included studies are found in Additional file 1.

## Discussion

The present literature review summarizes the evidence on ESWT in BME. It was found that several studies report quick improvements compared to other treatment options. The results were consistent independently of study type and study quality. This was also confirmed by the meta-analysis of eligible studies. Generally, there were significantly greater improvements of pain and patient reported function in patients being treated with ESWT both in short- and mid-term as well as long-term outcomes. No previous reviews on this topic were found. However, reviews on more specific clinical entities have also shown favourable outcomes after treatment with ESWT. Low-energy ESWT was shown to be an effective treatment for chronic medial tibial stress syndrome [21]. A review by Furia et al. concluded that shock wave therapy facilitates bone healing by stimulating angiogenesis and osteogenesis in disorders such as nonunions, avascular necrosis, and delayed healing of stress fractures [22].

In a review on the treatment of osteonecrosis of the femoral head, the authors concluded that pain and function may improve through ESWT, especially in early stages. However, they did not report outcomes on BME [23]. A network meta-analysis [24] showed that treatment failure rate was lowest when treated with core decompression plus cryotherapy. However, ESWT was best at improving HHS [24].

### Surgery vs. ESWT

Shock wave treatment has been debated as an alternative to surgery mostly in BME of the hip. This is particularly interesting because ESWT has only a few side effects like bruising and swelling. Surgery such as bone decompression on the contrary involves risks such as fracture and surgical site infections [7]. Therefore, it remains questionable whether surgery is too invasive for a self-limiting disease [1, 7]. Additionally, the cost of ESWT is less than surgery and results are often better [7]. Similarly, Furia et al. [22] concluded that shock wave therapy is an alternative to surgery both being cheaper and yielding fewer complications while achieving comparable success rates.

Chen et al. [15] even reported more favourable results with ESWT compared to THA, but the hips treated with ESWT usually presented earlier stages (ARCO stages I–III) of osteonecrosis of the femoral head than those treated with THA (ARCO stages III or IV). In addition, time between the two treatments ranged from 6 to 36 months and THA was the first treatment in all but 1 patient [15]. This may account for some of the differences. Adding to the favourable results in earlier stages, Vulpiani et al. [14] showed that ESWT in ARCO stages I and II may help to prevent progression of the area of avascular necrosis and to alleviate pain.

### ESWT vs. bisphosphonates and analogues of prostacyclin

Another established therapy for BME is the administration of bisphosphonates or analogues of prostacyclin. A study on treating avascular necrosis of the hip with alendronate found improvements in pain and hip range of motion as well as reductions of BME at 1 year follow-up [25]. Baier et al. compared intravenous bisphosphonate and prostacyclin treatment for BME of the knee and foot. They found similar improvements after 1 year; however, patients treated with prostacyclin showed quicker improvements [26].

There are not many trials directly comparing bisphosphonates with ESWT. However, one randomized study found greater and earlier improvements with ESWT than with alendronate and alprostadil [1]. This was also confirmed by another retrospective trial, which however administered alprostadil to both groups [4]. Oral alendronate was not able to further improve the clinical results when added to ESWT [12, 13].

When treated with ESWT, patients often experience marked improvements at1 months already, with bisphosphonates or prostacyclin usually not showing greater effects until 3 months [1].

There are multiple pathologies that display BME on MRI examination and there is ongoing debate about the connectedness of some of these. For example, it is not clear whether BME syndrome of the hip is an early stage of non-traumatic avascular necrosis. Some argue against this theory [7]. BME syndrome of the hip is accepted as a standalone clinical entity though progression to avascular necrosis has been described [2]. Further entities where BME is present are osteitis pubis, Kienböck’s disease, and BME of the knee associated with osteoarthritis. BME has also been observed in chronic plantar fasciitis [27], an entity that is often successfully treated with ESWT.

### Limitations

Many pathologies that display BME on MRI were included in this review. However, it is not clear if the effects observed in these studies are applicable to the treatment of BME in general. Although there seems to be a good responsiveness to ESWT, it is unknown if BME in some locations may behave differently. Future studies will have to show the treatment effects of ESWT in pathologies that have not been studied in the reviewed literature.

This systematic review found only very slight high-quality evidence on the treatment of BME with ESWT. In total, there were only two randomized-controlled studies and four non-randomized prospective studies and the overall heterogeneity was high. Therefore, while the general results are rather favourable across all types of studies, the general level of evidence is weak.

## Conclusion

Based on the available evidence, ESWT is a promising therapeutic approach to the treatment of BME that may even serve as an alternative to surgical treatment. Generally, there is a quick and marked improvement in pain and function after only a few sessions. Additional treatment with bisphosphonates and prostaglandin inhibitors does not seem to improve the outcome. The consistent outcomes across several pathologies may allow generalizing these beneficial effects of ESWT to bone marrow oedema in other locations. However, more high-quality, randomized studies, with distinct homogeneity between study designs, are needed to build upon the existing evidence for ESWT.

## Supplementary Information

**Additional file 1.** Table of studies which met eligibility criteria and risk of bias.

**Additional file 2.** PRISMA checklist.

**Additional file 3.** PROSPERO Intervention protocol.

## Data Availability

All data generated or analysed during this study are either included in this published article or its supplementary information files.

## Abbreviations

BME: Bone marrow oedema
ESWT: Extracorporeal shock wave therapy
HHS: Harris Hip Score
MRI: Magnetic resonance imaging
THA: Total hip arthroplasty
VAS: Visual analogue scale
WMD: Weighted mean difference
WOMAC: Western Ontario and McMaster Universities Osteoarthritis Index
