# Supplementary material for: The use of extracorporeal shock wave therapy for the treatment of bone marrow oedema — a systematic review and meta-analysis
Source: J Orthop Surg Res. 2021 Jun 9;16:369. doi: 10.1186/s13018-021-02484-5 (PMC8188716; doi:10.1186/s13018-021-02484-5)
Supplement: Supplementary file 1 — Additional file 1. Table of studies which met eligibility criteria and risk of bias. [file 13018_2021_2484_MOESM1_ESM.docx]

Table 1: Overview and risk of bias assessment of included randomized controlled trials.

| Study | n | Risk of bias (Downs and Black Score) | Groups | Follow up | Diagnosis | Results |
| --- | --- | --- | --- | --- | --- | --- |
| Gao, Sun, Li, Guo, Wang, Cheng, Yue, et al., 2015 (1) | 40 (female = 20, male = 20) | 23 | Partial weight‒bearing and walking aids for 6 weeks as well as analgesics on demand for both groups.  1. ESWT (2 sessions one week apart, energy flux density > 0.44 mJ/mm^2^,  3000 – 4000 impulses at  2 – 3 Hz  2. Alendronate 70 mg/week and Alprostadil 10 µg/days. | 1, 3, 6, 12 months  MRI only at 6 and 12 months | Primary bone marrow edema syndrome of the knee (excluded avascular necrosis and osteoarthritis grade 3 and 4) | Visual analogue scale, WOMAC score, SF-36 with greater and earlier improvements in group 1  (p < 0.05).  Significantly more patients in ESWT with reduction or complete resolution of edema on MRI at 6 months |
| Schöberl et al., 2017 (2) | 44 | 26 | Both groups had intensive rehabilitation program  1. ESWT: 3 sessions at weekly intervals with 1500 impulses with 15 – 21 Hz  2. sham ESWT | 1, 3, 12 months | Osteitis pubis | Visual analogue scale and Hip disability and Osteoarthritis Outcome Score showed significantly greater improvements in group 1 vs. group 2 after 1 and 3 months.  MRI findings were not significantly different  return-to-football was significantly earlier in group 1 (73.2 days) than in group 2 (102.6 days) |

SF-36: Short Form (36) Health Survey; WOMAC: Western Ontario and McMaster Universities Osteoarthritis Index; MRI: magnetic resonance imaging; ESWT: extracorporeal shock wave therapy

Table 2: Overview and risk of bias assessment of included prospective trials.

| Study | | n | Risk of bias (Downs and Black Score) | | Groups | | Follow up | | Diagnosis | | Results | |
| --- | --- | --- | --- | --- | --- | --- | --- | --- | --- | --- | --- | --- |
| d’Agostino, Romeo, Lavanga, Pisani, & Sansone, 2014 (3) | 20 (female = 8, male = 12) | | | 18 | | 2 sessions of ESWT at 48 h interval  4.000 shots at 0.5 mJ/mm^2^ | | 2, 3, 6 and 15.5 months  MRI at 2 and 6 months | | Bone marrow edema syndrome of the hip (ARCO stage I) | | HHS improved significantly from 39.08 ± 19.66 to 81.28 ± 10.8 after 2 months and 95.06 ± 4.53 after 6 months.  MRI showed regression of mean edema area from 981.9 ± 453.2 mm² to 469.5 ± 306.8 mm² at 2 months and 107.8 ± 248.1 mm² at  6 months. |
| Hsu et al., 2010 (4) | | 68 (105 hips) | 20 | | 1. ESWT + hyperbaric oxygen therapy + oral alendronate (12 month)  2. ESWT alone (single treatment, general anaesthesia, 6000 impulses, 0.62 mJ/mm² | | 1, 3, 6, 12 months, then yearly | | Osteonecrosis of the femoral head | | No significant difference between ESWT alone and combined treatment. | |
| Vulpiani et al., 2012 (5) | | 36 (female = 13, male = 23) | 22 | | 4 sessions, 2.400 impulses at 0.50 mJ/mm², at 48 – 72 h intervals | | 3, 6, 12 and then 24 months | | Avascular necrosis of femoral head ARCO stage I, II and III | | HHS  Stage I had fastest improvements, VAS 6.7 to 2.6 at 6 months, HHS from 42.5 to 89.8 at 6 months.  Stage III improved from VAS 7.2 to 2.6 at 24 months, but 10 converted to THA | |
| Wang et al., 2008 (6) | | 48 (female = 15, male = 33) | 20 | | One session of 6,000 impulses of ESWT at 0.62 mJ/mm²  1. ESWT only  2. EWST + alendronate 70 mg once weekly for 1 year | | 1, 3, 6 and 12 months, then once a year | | Osteonecrosis of the Femoral Head (ARCO Stage I, II, and III) | | significant improvements in pain and HHS.  No significant difference between groups.  THA in 10 % of both groups  MRI showed significant reduction in BME after treatment, no difference between groups. | |

BME: Bone marrow edema; ESWT: Extracorporeal shock wave therapy; HHS: Harris Hip score; MRI: Magnetic resonance imaging; THA: total hip arthroplasty; ARCO: Association Research Circulation Osseous

Table 3: Overview and risk of bias assessment of included retrospective studies.

| Study | n | Risk of bias (Downs and Black Score) | Groups | Follow up | Diagnosis | Results |
| --- | --- | --- | --- | --- | --- | --- |
| Chen et al., 2009 (7) | 17 (female = 3, male = 14) | 15 | THA in late stages of Osteonecrosis of the Femoral Head and ESWT in the hip with earlier stage (average time between THA and ESWT was 17.3 months).  ESWT: 6.000 impulses with 0.62 mJ/mm² | 41‒44.3 months | Bilateral osteonecrosis of the femoral head | 13 patients rated ESWT better than THA.  4 patients reported comparable results between THA and ESWT, and none graded THA better than ESWT.  VAS and Harris hip score with greater improvements in ESWT, MRI showed significant reduction of BME after ESWT. |
| D’Agostino, Romeo, Amelio, & Sansone, 2011 (8) | 22 patients (female = 6, male = 16) | 15 | Splinting of affected wrist  3 sessions of ESWT, 4000 shocks with local anesthetic, 0.35 ‒0.40 mJ/mm² at 4 Hz | 60, 180 days and 1 year | Kienböck’s disease  Lichtman Stage I-IIIb | VAS improved from 8.25 to 3.73 at 60 days.  Range of motion improved at 60 days.  Both VAS and range of motion slightly deteriorated after 60 days until 1 year.  MRI showed noticeable reduction in bone marrow edema in all patients. |
| Gao, Sun, Li, Guo, Kush, et al., 2015 (9) | 46 (female = 22, male = 24) | 21 | Both groups received 70 mg of alendronate weekly and 10 μg of Alprostadil each day for 14 days  1. High-energy ESWT  2 series of 3 treatments, 3 to 4 levels, and 2000 to 3000 impulses, each admin- istered at 0.50 mJ/mm^2^  2. Femoral head core decompression | 6 months | Intractable bone marrow edema syndrome of the hip | Improvements in VAS were significantly greater for ESWT.  ESWT resumed daily activities significantly earlier  full resolution of symptoms occurred significantly earlier in ESWT. |
| Gao, Sun, Li, Guo, Wang, Cheng, & Wang, 2015 (10) | 335 (female = 106, male = 229) | 19 | Two session ESWT with 3000 – 4000 impulses at 2‒3Hz with> 0.44mJ/mm²  70 mg alendronate for 12 months | 3, 6, and 12 months | Osteonecrosis of the Femoral Head (ARCO Stage I, II, and III) | VAS from 6.8 ± 3.7 to 1.0 ± 2.1  HHS from 69.4 ± 14.7 to 90.9 ± 11.4  MRI: significant reduction in BME after treatment. |
| Kang et al., 2018 (11) | 126 (female = 71, male= 55)  Retrospective | 18 | Both groups Alprostadil injections 10 µg 1x/d for 2 weeks  1. ESWT (2x, weekly, 3000 ‒4000 impulses, 0,44 mJ/mm²  2. 70 mg alendronate 1x/week | 23.5 month | BME, Osteoarthritis of the knee | Greater and earlier improvements in pain and WOMAC in group 1 . |
| Maki et al., 2017 (12) | 23 (female = 13, male = 10) | 12 | First conservative treatment including  stretching, nonsteroidal anti-inflammatory drugs, bracing, and steroid injections for 3 months.  Then 1 or 2 sessions of ESWT with 3800 rounds,  1 shock = 0.03 – 0.36 mJ/mm^2^ | 0 and6 months | Refractory plantar fasciitis | VAS improved from 74.1 ±25.3 to28.5 ± 24.4 mm.  Japanese Society for Surgery of the Foot ankle-hind foot scale improved from 70.3 ± 0.5 to 88.6 ± 9.1 points.  BME of the calcaneus was seen on MRI in 11 feet before and in 4 feet after ESWT |
| Sansone, Romeo, & Lavanga, 2017 (13) | 86 (female = 54, male =32) | 23 | 1. 3 sessions ESWT, every third week (2000 shots, 0.22 ‒ 0.43 mJ/mm², 4 Hz)  2. 31 patients, lived too far from hospital so only received protected weight‒bearing and analgesics | 3, 6, 18 months,  control only 6, 18 months | BME syndrome of the medial compartment of the knee | Western Ontario and McMaster Universities Osteoarthritis Index after 18 months improved by 34.9 in ESWT vs. 11.6 in control group (p < 0.001).  VAS after 18 months improved by 6.9 in ESWT vs. 3.2 in control (p<0,001); 53 % in ESWT pain-free.  MRI: area of BME after 6 months reduced by 88 % in ESWT vs. 41 % in control  (p < 0.001) and  already marked improvements in ESWT at  3 months.  No side effects. |
| Vitali et al., 2018 (14) | 56 (female = 39, male = 17) | 19 | 1. ESWT once a week, 3 sessions with 4000 shots at an energy flux density of 0.55mJ/mm^2^, crutches, no medication  2. control: 28 patients unable to come to institution because of distance, patients who did not tolerate the therapy, and patients who refused to undergo ESWT; analgesics and partial weight‒bearing | 1 and 4 months | BME of the medial condyle of the knee. | VAS, Knee Society Score clinical and functional scores at 1 and 4 months improved significantly greater in ESWT than in control.  Significantly greater improvement of edema area on MRI at 4 months in ESWT group. |
| Xie et al., 2018 (15) | 31 | 17 | ESWT with a total of 4000 impulses at 26 kV and 4 Hz  no to limited weight‒bearing in the first 3 months | 130.6 months | Non‒traumatic osteonecrosis of the femoral head ARCO stages I ‒ III | VAS, HHS significantly improved at 10 year follow‒up 🡪 Improvement of BME was seen in 30/43 hips |

BME: Bone marrow edema; ESWT: Extracorporeal shock wave therapy; HHS: Harris Hip score; MRI: Magnetic resonance imaging; THA: total hip arthroplasty; VAS: Visual analogue scale.

**References**

1. Gao F, Sun W, Li Z, Guo W, Wang W, Cheng L, et al. Extracorporeal shock wave therapy in the treatment of primary bone marrow edema syndrome of the knee: a prospective randomised controlled study. BMC Musculoskelet Disord. 2015 Dec;16:379.

2. Schöberl M, Prantl L, Loose O, Zellner J, Angele P, Zeman F, et al. Non-surgical treatment of pubic overload and groin pain in amateur football players: a prospective double-blinded randomised controlled study. Knee Surgery, Sport Traumatol Arthrosc. 2017 Jun 1;25(6):1958–66.

3. d’Agostino C, Romeo P, Lavanga V, Pisani S, Sansone V. Effectiveness of extracorporeal shock wave therapy in bone marrow edema syndrome of the hip. Rheumatol Int. 2014 Nov;34(11):1513–8.

4. Hsu S-L, Wang C-J, Lee MS-S, Chan Y-S, Huang C-C, Yang KD. Cocktail therapy for femoral head necrosis of the hip. Arch Orthop Trauma Surg. 2010 Jan;130(1):23–9.

5. Vulpiani MC, Vetrano M, Trischitta D, Scarcello L, Chizzi F, Argento G, et al. Extracorporeal shock wave therapy in early osteonecrosis of the femoral head: Prospective clinical study with long-term follow-up. Arch Orthop Trauma Surg. 2012 Apr;132(4):499–508.

6. Wang C-J, Wang F-S, Yang KD, Huang C-C, Lee MS-S, Chan Y-S, et al. Treatment of osteonecrosis of the hip: comparison of extracorporeal shockwave with shockwave and alendronate. Arch Orthop Trauma Surg [Internet]. 2008 Sep [cited 2020 Mar 17];128(9):901–8. Available from: http://www.ncbi.nlm.nih.gov/pubmed/18060419

7. Chen J-M, Hsu S-L, Wong T, Chou W-Y, Wang C-J, Wang F-S. Functional outcomes of bilateral hip necrosis: total hip arthroplasty versus extracorporeal shockwave. Arch Orthop Trauma Surg [Internet]. 2009 Jun [cited 2020 Mar 17];129(6):837–41. Available from: http://www.ncbi.nlm.nih.gov/pubmed/19165494

8. D’Agostino C, Romeo P, Amelio E, Sansone V. Effectiveness of ESWT in the treatment of Kienböck’s disease. Ultrasound Med Biol [Internet]. 2011 Sep [cited 2020 Mar 3];37(9):1452–6. Available from: http://www.ncbi.nlm.nih.gov/pubmed/21767905

9. Gao F, Sun W, Li Z, Guo W, Kush N, Ozaki K. Intractable bone marrow edema syndrome of the hip. Orthopedics. 2015 Apr;38(4):e263-70.

10. Gao F, Sun W, Li Z, Guo W, Wang W, Cheng L, et al. High-Energy Extracorporeal Shock Wave for Early Stage Osteonecrosis of the Femoral Head: A Single-Center Case Series. Evid Based Complement Alternat Med. 2015;2015:468090.

11. Kang S, Gao F, Han J, Mao T, Sun W, Wang B, et al. Extracorporeal shock wave treatment can normalize painful bone marrow edema in knee osteoarthritis: A comparative historical cohort study. Medicine (Baltimore). 2018 Feb;97(5):e9796.

12. Maki M, Ikoma K, Kido M, Hara Y, Sawada K, Ohashi S, et al. Magnetic resonance imaging findings of chronic plantar fasciitis before and after extracorporeal shock wave therapy. Foot (Edinb). 2017 Dec;33:25–8.

13. Sansone V, Romeo P, Lavanga V. Extracorporeal Shock Wave Therapy Is Effective in the Treatment of Bone Marrow Edema of the Medial Compartment of the Knee: A Comparative Study. Med Princ Pract. 2017;26(1):23–9.

14. Vitali M, Naim Rodriguez N, Pedretti A, Drossinos A, Pironti P, Di Carlo G, et al. Bone Marrow Edema Syndrome of the Medial Femoral Condyle Treated With Extracorporeal Shock Wave Therapy: A Clinical and MRI Retrospective Comparative Study. Arch Phys Med Rehabil. 2018 May;99(5):873–9.

15. Xie K, Mao Y, Qu X, Dai K, Jia Q, Zhu Z, et al. High-energy extracorporeal shock wave therapy for nontraumatic osteonecrosis of the femoral head. J Orthop Surg Res. 2018 Feb;13(1):25.
