# Supplementary material for: The use of extracorporeal shock wave therapy for the treatment of bone marrow oedema — a systematic review and meta-analysis
Source: J Orthop Surg Res. 2021 Jun 9;16:369. doi: 10.1186/s13018-021-02484-5 (PMC8188716; doi:10.1186/s13018-021-02484-5)
Supplement: Supplementary file 3 — Additional file 3. PROSPERO Intervention protocol. [file 13018_2021_2484_MOESM3_ESM.pdf]

Systematic review

*Fields that have an **asterisk (*)** next to them means that they **must be answered. Word limits** are provided for each section. You will be unable to submit the form if the word limits are exceeded for any section. Registrant means the person filling out the form*.

1. * Review title.

Give the title of the review in English

Use of extracorporeal shock wave therapy for the treatment of bone marrow edema – a systematic review

2. Original language title.

For reviews in languages other than English, give the title in the original language. This will be displayed with the English language title.

3. * Anticipated or actual start date.

Give the date the systematic review started or is expected to start.

01/06/2020

4. * Anticipated completion date.

Give the date by which the review is expected to be completed.

28/01/2021

5. * Stage of review at time of this submission.

Tick the boxes to show which review tasks have been started and which have been completed.
Update this field each time any amendments are made to a published record.

Reviews that have started data extraction (at the time of initial submission) are not eligible for inclusion in PROSPERO.
If there is later evidence that incorrect status and/or completion date has been supplied, the published PROSPERO record will be marked as retracted.

This field uses answers to initial screening questions. It cannot be edited until after registration.

The review has not yet started: No

Review stage	Started	Completed
Preliminary searches	Yes	Yes
Piloting of the study selection process	Yes	Yes
Formal screening of search results against eligibility criteria	Yes	Yes
Data extraction	Yes	Yes
Risk of bias (quality) assessment	Yes	Yes

Review stage	Started	Completed
Data analysis	Yes	Yes

Provide any other relevant information about the stage of the review here.

6. * Named contact.

The named contact is the guarantor for the accuracy of the information in the register record. This may be any member of the review team.

Juliane Wieber

Email salutation (e.g. "Dr Smith" or "Joanne") for correspondence:
Ms Wieber

7. * Named contact email.

Give the electronic email address of the named contact.

j.wieber@lanserhof.com

8. Named contact address

PLEASE NOTE this information will be published in the PROSPERO record so please do not enter private information, i.e. personal home address

Give the full institutional/organisational postal address for the named contact.

Ms Juliane Wieber

LANS Medicum (Center for Sports and Regenerative Medicine)

Stephansplatz 5

20354 Hamburg

9. Named contact phone number.

Give the telephone number for the named contact, including international dialling code.

+49 1784778644

10. * Organisational affiliation of the review.

Full title of the organisational affiliations for this review and website address if available. This field may be completed as 'None' if the review is not affiliated to any organisation.

LANS Medicum (Center for Sports and Regenerative Medicine)

Organisation web address:
https://www.lanserhof.com/en

11. * Review team members and their organisational affiliations.

Give the personal details and the organisational affiliations of each member of the review team. Affiliation refers to groups or organisations to which review team members belong.
NOTE: email and country now MUST be entered for each person, unless you are amending a published record.

Ms Juliane Wieber. LANS Medicum (Center for Sports and Regenerative Medicine)
Mr Jonathan Häußer. LANS Medicum (Center for Sports and Regenerative Medicine)

Dr Philip Catalá-Lehnen. LANS Medicum (Center for Sports and Regenerative Medicine)

12. * Funding sources/sponsors.

Details of the individuals, organizations, groups, companies or other legal entities who have funded or sponsored the review.

LANS Medicum Hamburg. no fundings provided

Juliane Wieber (ORCID-ID:0000-0002-2904-9305)

Grant number(s)

State the funder, grant or award number and the date of award

13. * Conflicts of interest.

List actual or perceived conflicts of interest (financial or academic).

None

14. Collaborators.

Give the name and affiliation of any individuals or organisations who are working on the review but who are not listed as review team members. **NOTE: email and country must be completed for each person, unless you are amending a published record.**

15. * Review question.

State the review question(s) clearly and precisely. It may be appropriate to break very broad questions down into a series of related more specific questions. Questions may be framed or refined using PI(E)COS or similar where relevant.

The question of this review was whether shock wave therapy is an effective treatment for improving pain and function in patients displaying BME and whether the effect of shock wave therapy is comparable to other conservative measures and surgery

16. * Searches.

State the sources that will be searched (e.g. Medline). Give the search dates, and any restrictions (e.g. language or publication date). Do NOT enter the full search strategy (it may be provided as a link or attachment below.)

Restriction by language: Sources not written in english or german language.
No restriction of publication period
Search date: 03.06.2020

17. URL to search strategy.

Upload a file with your search strategy, or an example of a search strategy for a specific database, (including the keywords) in pdf or word format. In doing so you are consenting to the file being made publicly accessible.

Or provide a URL or link to the strategy. Do NOT provide links to your search **results**.

https://www.crd.york.ac.uk/PROSPEROFILES/201719_STRATEGY_20210128.pdf

Yes I give permission for this file to be made publicly available

18. * Condition or domain being studied.

Give a short description of the disease, condition or healthcare domain being studied in your systematic review.

Treatment of Extracorporeal shock wave therapy (ESWT) in Bone marrow edema (BME). BME may occur in many different locations. BME presents as a painful increase in interstitial fluid. It is most likely a vascular reaction to external or internal

disorders.

19. * Participants/population.

Specify the participants or populations being studied in the review. The preferred format includes details of both inclusion and exclusion criteria.

Inclusion criteria:
- patients with bone marrow edema or disease involving bone marrow edema
- studies reporting pain and functional outcomes in the short, middle, and long term as well as changes on MRI

Exclusion criteria:
- patients with fatigue induced fractures
- publications in languages other than German or English
- case reports and conference abstracts

20. * Intervention(s), exposure(s).

Give full and clear descriptions or definitions of the interventions or the exposures to be reviewed. The preferred format includes details of both inclusion and exclusion criteria.

Extracorporeal shock wave therapy (ESWT) is an option for conservative treatment in bone marrow edema. A growing body of evidence has shown its effectiveness in multiple orthopedic conditions.The mechanism of action is not entirely clear. Generally speaking, shock wave therapy promotes a tissue’s self-healing capabilities . In bone tissue this involves stimulation of osteoblasts and periosteal cells, differentiation of stem cells, and increased secretion of nitric oxide synthase (eNOS) and vascular endothelial growth factor (VEGF) thus leading to increased neovascularization. Additionally, the periosteum is stimulated and osteoclasts activity is reduced.

Inclusion: pain and functional outcomes in the short, middle, and long term as well as changes on MRI
Exclusion: Case reports, conference abstracts and publications not written in English or German

21. * Comparator(s)/control.

Where relevant, give details of the alternatives against which the intervention/exposure will be compared (e.g. another intervention or a non-exposed control group). The preferred format includes details of both inclusion and exclusion criteria.

Surgery. Patients who get surgery for bone marrow edema.
Surgery is the last resort and the most common surgical technique is core decompression. This usually leads to significant improvements after 4 weeks. However, surgery is prone to complications such as wound infections, fracture, and hematoma. With BME being a self-limiting condition, some consider surgery too invasive.
Patients which got bisphosphonates and prostacyclin analogues as a comparator variable

22. * Types of study to be included.

Give details of the study designs (e.g. RCT) that are eligible for inclusion in the review. The preferred format includes both inclusion and exclusion criteria. If there are no restrictions on the types of study, this should be stated.

We will include randomised (controlled) trials to assess the beneficial effects of the treatments, and will supplement these with observational studies (including cohort and case–control studies) for the assessment of outcomes after extracorporal shockwave therapy and surgery. Ongoing randomized controlled studies or studies without complete data for analysis will be excluded.

23. Context.

Give summary details of the setting or other relevant characteristics, which help define the inclusion or exclusion criteria.

24. * Main outcome(s).

Give the pre-specified main (most important) outcomes of the review, including details of how the outcome is defined and measured and when these measurement are made, if these are part of the review inclusion criteria.

The review wants to achieve knowledge in which way there is a therapeutical benefit of ESWT compared to surgery and other conservative treatment options.

* Measures of effect

The main outcomes are pain and function measured on a visual analogue scale and by patient reported outcome questionaires (Hip Harris score, Western Ontario and McMaster Universities Osteoarthritis Index, Hip Osteoarthritis Outcome Score, Knee Society Scale). All outcome criteria in the short, middle and long term are of interest.

25. * Additional outcome(s).

List the pre-specified additional outcomes of the review, with a similar level of detail to that required for main outcomes. Where there are no additional outcomes please state ‘None’ or ‘Not applicable’ as appropriate to the review

Additional outcomes are changes of bone marrow edema area on MR imaging in the short middle and long term.

* Measures of effect

none

26. * Data extraction (selection and coding).

Describe how studies will be selected for inclusion. State what data will be extracted or obtained. State how this will be done and recorded.

Titles and abstracts will be screened independently by two authors for relevant publications. One researcher finally checks the decision. Articles that reporte pain and functional outcomes in the short, middle, and long term as well as changes on MRI will be included. Publications not written in English or German will be excluded. Of all the remaining publications full texts will be retrieved. If there is disagreement about the inclusion of certain studies the situation should be resolved by consensus. Data on pain, function and changes on MRI imaging will be extracted from included papers. One researcher will extract data and an other person check the extracted data. The findings will be reported according to the PRISMA guidelines.

27. * Risk of bias (quality) assessment.

State which characteristics of the studies will be assessed and/or any formal risk of bias/quality assessment tools that will be used.

For the assessment of study quality we will use a modified version of the Downs and Black checklist (Downs & Black, 1998) as shown by (Korakakis, Whiteley, Tzavara, & Malliaropoulos, 2018). If there is disagreements between the review authors over the risk of bias in particular studies a third review author will be involved and the risk of bias will be dicussed until solution.

28. * Strategy for data synthesis.

Describe the methods you plan to use to synthesise data. This **must not be generic text** but should be **specific to your review** and describe how the proposed approach will be applied to your data.
If meta-analysis is planned, describe the models to be used, methods to explore statistical heterogeneity, and software package to be used.

It should be present tables to provide a narrative synthesis of the findings from the included studies, They will be strucrured as: Study (author, titel); type of intervention, target population characteristics, type of outcome and intervention content, risk of bias index

29. * Analysis of subgroups or subsets.

State any planned investigation of ‘subgroups’. Be clear and specific about which type of study or participant will be included in each group or covariate investigated. State the planned analytic approach.

Subgroup analyses will be done for participants having different indications for ESWT treatment, at different follow-ups, and with different controls. This is a systemic review including
qualitative and quantitative synthesis and it is not possible to specify the
groups in advance

30. * Type and method of review.

Select the type of review, review method and health area from the lists below.

Type of review

Cost effectiveness	No
Diagnostic	No
Epidemiologic	No
Individual patient data (IPD) meta-analysis	No
Intervention	Yes
Meta-analysis	No
Methodology	No
Narrative synthesis	No
Network meta-analysis	No
Pre-clinical	No
Prevention	No
Prognostic	No
Prospective meta-analysis (PMA)	No
Review of reviews	No
Service delivery	No
Synthesis of qualitative studies	No
Systematic review	Yes
Other	No

Health area of the review

Alcohol/substance misuse/abuse	No
Blood and immune system	No
Cancer	No
Cardiovascular	No
Care of the elderly	No
Child health	No
Complementary therapies	Yes

COVID-19	No
Crime and justice	No
Dental	No
Digestive system	No
Ear, nose and throat	No
Education	No
Endocrine and metabolic disorders	No
Eye disorders	No
General interest	No
Genetics	No
Health inequalities/health equity	No
Infections and infestations	No
International development	No
Mental health and behavioural conditions	No
Musculoskeletal	Yes
Neurological	No
Nursing	No
Obstetrics and gynaecology	No
Oral health	No
Palliative care	No
Perioperative care	No
Physiotherapy	No
Pregnancy and childbirth	No
Public health (including social determinants of health)	No
Rehabilitation	Yes
Respiratory disorders	No
Service delivery	No

Skin disorders	No
Social care	No
Surgery	No
Tropical Medicine	No
Urological	No
Wounds, injuries and accidents	No
Violence and abuse	No

31. Language.

Select each language individually to add it to the list below, use the bin icon to remove any added in error.

English

There is an English language summary.

32. * Country.

Select the country in which the review is being carried out. For multi-national collaborations select all the countries involved.

Germany

33. Other registration details.

Name any other organisation where the systematic review title or protocol is registered (e.g. Campbell, or The Joanna Briggs Institute) together with any unique identification number assigned by them.
If extracted data will be stored and made available through a repository such as the Systematic Review Data Repository (SRDR), details and a link should be included here. If none, leave blank.

34. Reference and/or URL for published protocol.

If the protocol for this review is published provide details (authors, title and journal details, preferably in Vancouver format)

Yes I give permission for this file to be made publicly available

35. Dissemination plans.

Do you intend to publish the review on completion?

Yes

36. Keywords.

Give words or phrases that best describe the review. Separate keywords with a semicolon or new line. Keywords help PROSPERO users find your review (keywords do not appear in the public record but are included in searches). Be as specific and precise as possible. Avoid acronyms and abbreviations unless these are in wide use.

Orthopaedic;

non-surgical treatment;
bone marrow edema;
extracorporeal shockwave therapy;
Osteonecrosis of the femoral head;
Osteitis pubis; Kienböck’s disease;
Plantar fasciitis

37. Details of any existing review of the same topic by the same authors.

If you are registering an update of an existing review give details of the earlier versions and include a full bibliographic reference, if available.

38. * Current review status.

Update review status when the review is completed and when it is published.
New registrations must be ongoing so this field is not editable for initial submission.

Review_Ongoing

39. Any additional information.

Provide any other information relevant to the registration of this review.

This review is being undertaken as possibly part of the planning for a randomised (controlled) trial to compare different types of therapy in comparison to ESWT.

40. Details of final report/publication(s) or preprints if available.

Leave empty until publication details are available OR you have a link to a preprint (NOTE: this field is not editable for initial submission).
List authors, title and journal details preferably in Vancouver format.
